# Serum GFAP and NfL as disease severity and prognostic biomarkers in patients with aquaporin-4 antibody-positive neuromyelitis optica spectrum disorder

**DOI:** 10.1186/s12974-021-02138-7

**Published:** 2021-05-01

**Authors:** Patrick Schindler, Ulrike Grittner, Johanna Oechtering, David Leppert, Nadja Siebert, Ankelien S. Duchow, Frederike C. Oertel, Susanna Asseyer, Joseph Kuchling, Hanna G. Zimmermann, Alexander U. Brandt, Pascal Benkert, Markus Reindl, Sven Jarius, Friedemann Paul, Judith Bellmann-Strobl, Jens Kuhle, Klemens Ruprecht

**Affiliations:** 1 Department of Neurology, Charité – Universitätsmedizin Berlin, corporate member of Freie Universität Berlin, Humboldt-Universität zu Berlin, and Berlin Institute of Health, Berlin, Germany; 2 Institute for Biometry and Clinical Epidemiology, Charité – Universitätsmedizin Berlin, corporate member of Freie Universität Berlin, Humboldt-Universität zu Berlin, and Berlin Institute of Health, Berlin, Germany; 3 Berlin Institute of Health (BIH), Berlin, Germany; 4 Neurologic Clinic and Policlinic, Departments of Medicine, Biomedicine and Clinical Research, University Hospital Basel, University of Basel, Basel, Switzerland; 5 NeuroCure Clinical Research Center, Charité – Universitätsmedizin Berlin, corporate member of Freie Universität Berlin, Humboldt-Universität zu Berlin, and Berlin Institute of Health, Berlin, Germany; 6 Experimental and Clinical Research Center, Max Delbrueck Center for Molecular Medicine and Charité – Universitätsmedizin Berlin, corporate member of Freie Universität Berlin, Humboldt-Universität zu Berlin, and Berlin Institute of Health, Berlin, Germany; 7 Department of Neurology, University of California San Francisco, San Francisco, CA USA; 8 Department of Neurology, University of California, Irvine, CA USA; 9 Clinical Trial Unit, Department of Clinical Research, University Hospital Basel, University of Basel, Basel, Switzerland; 10 Clinical Department of Neurology, Medical University of Innsbruck, Innsbruck, Austria; 11 Molecular Neuroimmunology Group, Department of Neurology, University of Heidelberg, Heidelberg, Germany

**Keywords:** Neuromyelitis optica spectrum disorder, Glial fibrillary acidic protein, Neurofilament light chain protein, Aquaporin-4 immunoglobulin G, Myelin oligodendrocyte glycoprotein immunoglobulin G, Biomarker, Serum

## Abstract

**Background:**

Neuromyelitis optica spectrum disorder (NMOSD) is a frequently disabling neuroinflammatory syndrome with a relapsing course. Blood-based disease severity and prognostic biomarkers for NMOSD are a yet unmet clinical need. Here, we evaluated serum glial fibrillary acidic protein (sGFAP) and neurofilament light (sNfL) as disease severity and prognostic biomarkers in patients with aquaporin-4 immunoglobulin (Ig)G positive (AQP4-IgG^+^) NMOSD.

**Methods:**

sGFAP and sNfL were determined by single-molecule array technology in a prospective cohort of 33 AQP4-IgG^+^ patients with NMOSD, 32 of which were in clinical remission at study baseline. Sixteen myelin oligodendrocyte glycoprotein IgG-positive (MOG-IgG^+^) patients and 38 healthy persons were included as controls. Attacks were recorded in all AQP4-IgG^+^ patients over a median observation period of 4.25 years.

**Results:**

In patients with AQP4-IgG^+^ NMOSD, median sGFAP (109.2 pg/ml) was non-significantly higher than in MOG-IgG^+^ patients (81.1 pg/ml; *p* = 0.83) and healthy controls (67.7 pg/ml; *p* = 0.07); sNfL did not substantially differ between groups. Yet, in AQP4-IgG^+^, but not MOG-IgG^+^ patients, higher sGFAP was associated with worse clinical disability scores, including the Expanded Disability Status Scale (EDSS, standardized effect size = 1.30, *p* = 0.007) and Multiple Sclerosis Functional Composite (MSFC, standardized effect size = − 1.28, *p* = 0.01). While in AQP4-IgG^+^, but not MOG-IgG^+^ patients, baseline sGFAP and sNfL were positively associated (standardized effect size = 2.24, *p* = 0.001), higher sNfL was only non-significantly associated with worse EDSS (standardized effect size = 1.09, *p* = 0.15) and MSFC (standardized effect size = − 1.75, *p* = 0.06) in patients with AQP4-IgG^+^ NMOSD. Patients with AQP4-IgG^+^ NMOSD with sGFAP > 90 pg/ml at baseline had a shorter time to a future attack than those with sGFAP ≤ 90 pg/ml (adjusted hazard ratio [95% confidence interval] = 11.6 [1.3–105.6], *p* = 0.03). In contrast, baseline sNfL levels above the 75^th^ age adjusted percentile were not associated with a shorter time to a future attack in patients with AQP4-IgG^+^ NMOSD.

**Conclusion:**

These findings suggest a potential role for sGFAP as biomarker for disease severity and future disease activity in patients with AQP4-IgG^+^ NMOSD in phases of clinical remission.

## Background

Neuromyelitis optica spectrum disorder (NMOSD) is a severe neuroinflammatory syndrome primarily affecting the optic nerves, spinal cord, and brainstem, with a relapsing and frequently disabling course, that is difficult to predict at the individual level [1, 2]. NMOSD is associated with immunoglobulin (Ig)G autoantibodies against the astrocytic water channel aquaporin-4 (AQP4-IgG) [3, 4]. AQP4-IgG causes an antibody-mediated astrocytopathy, thereby playing a key role in the immunopathogenesis of NMOSD [5, 6].

Current diagnosis of NMOSD is based on the International Panel for NMO Diagnosis (IPND) criteria [7]. Among patients meeting the IPND criteria, ~ 75% have AQP4-IgG [8]. Among the remaining AQP4-IgG seronegative patients, ~ 40% have IgG against myelin oligodendrocyte glycoprotein (MOG-IgG), while ~ 60% are AQP4-IgG/MOG-IgG double seronegative [8]. MOG-IgG has been associated with inflammatory demyelination and defines a disease entity now termed MOG antibody disease (MOGAD), whose clinical presentation partially overlaps with NMOSD as defined by the IPND criteria [6, 9–11]. However, whereas astrocytes are considered the primary autoantibody target in AQP4-IgG^+^ NMOSD, in MOGAD, oligodendrocytes are considered the primary autoantibody target, as MOG is expressed on oligodendrocytes [9, 11].

Given that relapse preventing treatment of AQP4-IgG^+^ NMOSD relies on immunotherapies with potentially relevant side effects, blood-based biomarkers for disease severity and future disease activity, that could help guiding treatment decisions in patients with AQP4-IgG^+^ NMOSD, are a yet unmet clinical need. Glial fibrillary acidic protein (GFAP) is an astrocytic intermediate filament [12], which is strongly elevated in cerebrospinal fluid (CSF) during attacks of NMOSD [13–16]. Neurofilament light chain protein (NfL) is a neuronal intermediate filament used as a biomarker for neuroaxonal damage [17]. Single-molecule array (Simoa) technology enables the ultrasensitive detection of both markers in serum [18, 19]. Employing Simoa technology, two recent studies, which each analyzed 33 patients with NMOSD, observed elevated serum GFAP (sGFAP) levels in patients with AQP4-IgG^+^ NMOSD, especially after recent attacks, and an association of sGFAP with the Expanded Disability Status Scale (EDSS), a clinical disability score [20, 21]. However, it is unclear whether those results, which were obtained in patients with rather active disease (35% and 65% of patients, respectively, with a recent relapse) [20, 21], may also apply to clinically stable patients. Furthermore, the role of sGFAP and serum NfL (sNfL) as prognostic biomarkers in patients with AQP4-IgG^+^ NMOSD remains to be defined in prospective cohorts.

Here, we performed a detailed investigation of sGFAP and sNfL as disease severity and prognostic biomarkers in a well-characterized prospective cohort of 33 patients with AQP4-IgG^+^ NMOSD. We also included MOG-IgG^+^ patients (*n* = 16) as specificity controls as well as healthy individuals (*n* = 38). AQP4-IgG^+^ patients were followed over a median observation period of 4.25 years. Altogether, the findings of this study suggest a potential role of sGFAP as biomarker for disease severity and future disease activity in patients with AQP4-IgG^+^ NMOSD in phases of clinical remission.

## Methods

### Participants

Patients were recruited at the Department of Neurology and the NeuroCure Clinical Research Center, Charité – Universitätsmedizin Berlin, between August 2015 and March 2018 and participate in an ongoing prospective longitudinal study of patients with NMOSD and related disorders. Inclusion criteria for the present investigation comprised age > 18 years, a diagnosis of AQP4-IgG^+^ NMOSD according to the 2015 IPND consensus criteria [7], or a diagnosis of MOG-IgG associated encephalomyelitis (herein referred to as “MOG-IgG^+^ patients”) according to the criteria of Jarius and colleagues [22]. All patients included in this study were Caucasians. At the baseline visit of the study, demographics and medical history, including prior attacks, were obtained and all patients underwent thorough neurological examination with assessment of the EDSS [23] by a trained EDSS rater. Additionally, the Timed 25-Foot Walk (T25-FW), 9-Hole Peg Test (9-HPT), and Paced Auditory Serial Addition Test (PASAT) with single digits every 3 s, composing the Multiple Sclerosis Functional Composite (MSFC) [24], were carried out. MSFC scores were calculated as previously described [25], using our cohort’s respective baseline means and standard deviations for Z-transformation. Patient sera were collected at the baseline visit, processed according to standard operating procedures and stored at − 80 °C. Applying exactly the same procedures, sera were collected from healthy controls (HC) recruited among hospital staff. In patients with NMOSD, all assessments and procedures were repeated at yearly follow-up visits. Patients with a relapse within ≤ 90 days before the baseline visit were considered to have active disease and patients with a relapse > 90 days before the baseline visit were considered to be in clinical remission.

### Laboratory procedures

AQP4-IgG and MOG-IgG were determined in serum using fixed cell-based assays (CBAs, Euroimmun, Lübeck, Germany), in-house fixed CBAs (Sven Jarius, University of Heidelberg, Germany) and in-house live CBAs (Markus Reindl, Medical University Innsbruck, Austria). Patients were classified as either AQP4-IgG^+^ or MOG-IgG^+^ if they had tested positive for AQP4-IgG or MOG-IgG at least once during their disease course. None of the patients were AQP4-IgG and MOG-IgG double positive. Coded serum samples were shipped on dry ice to the Department of Neurology, University of Basel, Switzerland, where sGFAP and sNfL were determined by Simoa (Quanterix, Lexington, MA, USA) by operators blinded to clinical data, as previously described [20, 26].

### Statistical analyses

Non-normally distributed variables (sGFAP, sNfL, time intervals, T25-FW) were log-transformed to meet the normal distribution assumption for use in parametric models. Differences of baseline sGFAP and sNFL in women and men were assessed by Mann-Whitney tests, and differences in sGFAP and sNfL between baseline and follow up by Wilcoxon signed-rank test.

Further group comparisons and association analyses were conducted using linear models, including age and, where applicable, interval since last attack before baseline as covariates. To provide comparable measures for the magnitude of investigated phenomena as well as to account for the dependency of null hypothesis significance tests on sample size, we calculated standardized measures of effect sizes [27]. To assess differences of baseline characteristics between groups (Table 1), we calculated absolute standardized mean difference values (SMD; R packages “tableone”). For association analyses, we calculated adjusted standardized effect sizes (SES; R package “emmeans”). Both SMD and SES are derived from mean differences in relation to the common standard deviation. Values of SMD or SES > 0.8 or < − 0.8 were considered as meaningful effect sizes, irrespective of the corresponding *p* value [28]. Accordingly, associations with an SES > 0.8 or < − 0.8 and a *p* value > 0.05 are reported as “non-significant associations.”

**Table 1:** Baseline demographic and clinical findings of AQP4-IgG^+^ patients, MOG-IgG^+^ patients and healthy controls

	AQP4-IgG^+^	MOG-IgG^+a^	Healthy Controls	SMD for AQP4-IgG^+^ vs. MOG-IgG^+^	SMD for AQP4-IgG^+^ vs. HC
Number, *n*	33	16^b^	38	–	–
age, years, mean (SD)	50 (14)	46 (15)	42 (13)	0.27	0.60
Female/male, n/n (% female)	30/3 (91)	10/6 (63)	31/7 (82)	0.71	0.27
Time from disease onset to baseline visit, months, median (IQR)	79 (52–108)	50 (10–148)	n.a.	0.04	–
Time from last attack prior to baseline visit to baseline visit, months, median (IQR)	26 (11–56)	8 (4–24)	n.a.	0.53	–
≥ 1 attack during previous year, *n* (%)	11 (33)	11 (69)	n.a.	0.76	–
Type of last attack, *n* (%)^c^				0.65	–
Optic neuritis	12 (36)	10 (63)	n.a.		
Myelitis	19 (58)	6 (37)	n.a.		
Brainstem encephalitis	1 (3)	0 (0)	n.a.		
EDSS, median (IQR)	4.0 (2.0–5.0)	2.5 (2.0–3.0)	n.a.	0.63	–
MSFC, mean (SD)	− 0.03 (0.69)	0.25 (0.58)	n.a.	0.44	–
9-HPT score, mean (SD)	0.0484 (0.0084)	0.0483 (0.0084)	n.a.	0.01	–
T25-FW, s, median (IQR)	5.3 (4.2– 6.2)	4.1 (3.1– 4.9)	n.a.	0.92	–
PASAT, median (IQR)	51 (33– 55)	54 (42– 58)	n.a.	0.31	–
Immunotherapy, *n* (%)	No: 4 (12) Any: 29 (88)	No: 4 (25) Any: 12 (75)	n.a.	0.34	–
	RTX: 20 (61) AZA: 6 (18)	RTX: 8 (50) AZA: 1 (6)	n.a.	–	–
	MMF: 1 (3) BEL: 1 (3) TCZ: 1 (3)	MMF: 1 (6) GLC: 2 (13)	n.a.	–	–
sGFAP (pg/ml), median (IQR)	109.2 (63.1– 154.8)	81.1 (58.2–116.9)	67.7 (56.6–90.7)	0.03	0.86
sNfL (pg/ml), median (IQR)	21.9 (16.6–41.4)	26.6 (15.9– 43.7)	19.2 (13.7– 29.4)	0.23	0.45

^a^In all MOG-IgG^+^ patients, presence of MOG-IgG^+^ was confirmed in at least two different assays

^b^Of the 16 MOG-IgG^+^ patients, 4 met the Wingerchuk 2015 criteria for AQP4-IgG^-^ NMOSD [7]

^c^One AQP4-IgG^+^ patient had sudden-onset gait impairment as leading symptom at last attack, not clearly attributable to either a brainstem or a myelon lesion

*9*-*HPT* 9-Hole Peg Test, *AQP4*-*IgG* aquaporin-4 immunoglobulin G, *AZA* azathioprine, *BEL* belimumab, *EDSS* Expanded Disability Status Scale, *GLC* glucocorticosteroids, *IQR* inter quartile range, *MMF* mycophenolate mofetil, *MOG*-*IgG* myelin oligodendrocyte protein immunoglobulin G, *MSFC* multiple sclerosis functional composite, *n* number, *n*.*a*. not applicable, *NMOSD* neuromyelitis optica spectrum disorder, *PASAT* paced auditory serial addition test, *RTX* rituximab, *s* seconds, *SD* standard deviation, *sGFAP* serum glial fibrillary acidic protein, *SMD* standardized mean difference, *sNfL* serum neurofilament light chain protein, *T25*-*FW* timed 25-foot walk, *TCZ* tocilizumab

To assess inter group differences of associations between sGFAP or sNFL and other parameters, interaction analyses were conducted [29]. To this end, the group variable and an interaction term of log-transformed baseline sGFAP or sNfL by group were additionally included in the model. Results of these analyses are reported as partial eta squared (η_p_^2^; R package “effectsize”), an adjusted measure of the size of the interaction effect, together with the respective *p* values.

Time to first attack was modeled using Cox proportional hazards regression. For Cox regression analyses, the observation period was calculated from the last attack prior to baseline. Cox regression analyses were adjusted for age and the interval between baseline visit and last prior attack. For Cox regression analyses, sNfL concentrations were converted into age adjusted sNfL percentiles derived from sNfL concentrations determined in a large group of healthy individuals using the same assay as in this study [30].

Statistical analyses were performed with R, version 3.6.2 (association analyses, calculation of effect size measures); Stata statistics software (Release 15, StataCorp LLC, College Station, TX, USA; testing the proportional hazards assumption for Cox regression); and IBM SPSS statistics software, version 25 (IBM, Armonk, NY, USA; all other statistics). Figures were created with GraphPad Prism, version 8.2.1 (GraphPad Software, San Diego, CA, USA; Fig. 2); IBM SPSS (Fig. 5); and R (all other figures).

*P* values < 0.05 were considered significant. Due to the exploratory character of this study, no correction for multiple testing was applied. All *p* values have to be interpreted with caution.

## Results

### Participants

Baseline demographic and clinical findings as well as sGFAP and sNfL values of AQP4-IgG^+^ and MOG-IgG^+^ patients and of HC are summarized in Table 1. Patients with AQP4-IgG^+^ NMOSD were on average 8 years older than HC. Only 1 of 33 (3 %) patients with AQP4-IgG^+^ NMOSD and 2 of 16 (13 %) MOG-IgG^+^ patients had encountered an attack within ≤ 90 days before the baseline visit. Thus, the vast majority of AQP4-IgG^+^ and MOG-IgG^+^ patients was in clinical remission at study inclusion with a median (interquartile range [IQR]) time since last attack of 26 (11–56) months in AQP4-IgG^+^ patients and 8 (4–24) months in MOG-IgG^+^ patients. The majority of AQP4-IgG^+^ (88 %) and MOG-IgG^+^ (75 %) patients was treated with immunotherapies at study baseline.

### Association of sGFAP and sNfL with age and gender

**Fig. 1:**
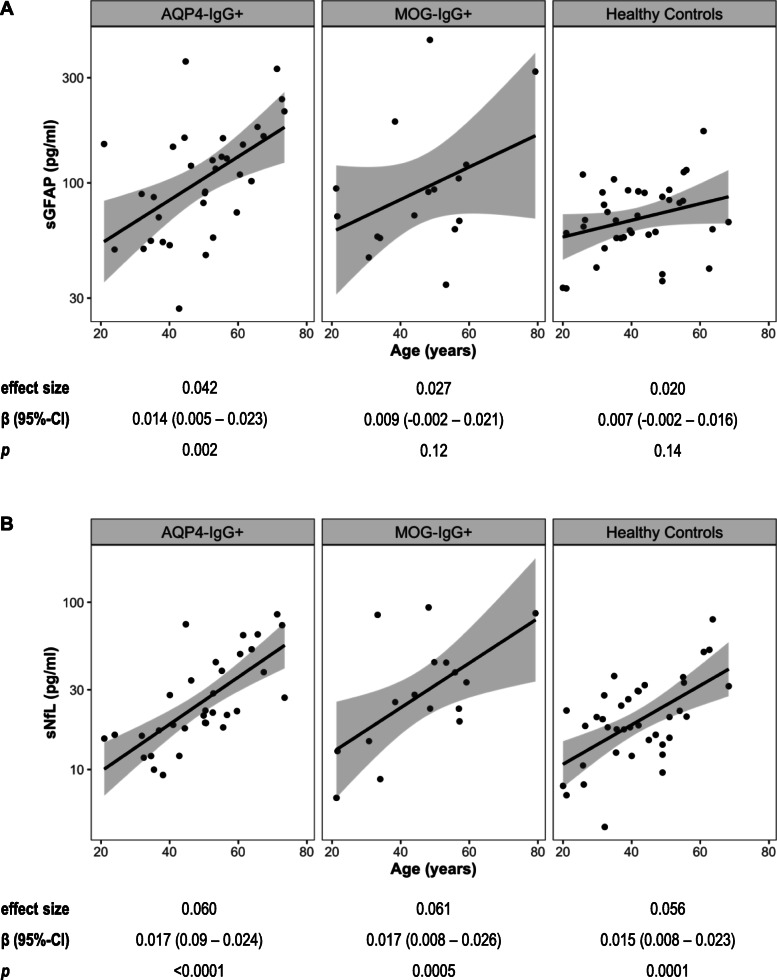
Association of sGFAP and sNfL with age. Association of sGFAP **a** and sNfL **b** with age of patients with AQP4-IgG^+^ NMOSD (*n* = 33), MOG-IgG^+^ patients (*n* = 16), and healthy controls (*n* = 38). Results of linear models using log-transformed values of sGFAP and sNfL are shown below the plots. *AQP4*-*IgG* aquaporin-4 immunoglobulin G, *β* regression coefficient, *CI* confidence interval, *effect size* standardized effect size, *MOG*-*IgG* myelin oligodendrocyte protein immunoglobulin G, *NMOSD* neuromyelitis optica spectrum disorder, *sGFAP* serum glial fibrillary acidic protein, *sNfL* serum neurofilament light chain protein

While higher sGFAP was associated with higher age in patients with AQP4-IgG^+^ NMOSD (SES = 0.042, *p* = 0.002), higher sGFAP was not significantly associated with higher age in MOG-IgG^+^ patients (SES = 0.027, *p* = 0.12) and HC (SES = 0.02, *p* = 0.14; interaction effect sGFAP by group η_p_^2^ = 0.02, *p* = 0.48; Fig. 1a). In contrast, higher sNfL was strongly associated with higher age in AQP4-IgG^+^ patients (SES = 0.06, *p* < 0.0001), MOG-IgG^+^ patients (SES = 0.061, *p* = 0.0005), and HC (SES = 0.056, *p* = 0.0001; interaction effect sNfL by group η_p_^2^ < 0.01, *p* = 0.96; Fig. 1b). Given the association of sGFAP and sNfL with age, all subsequent linear models and Cox regression analyses were adjusted for age.

There were no relevant differences in median sGFAP and sNfL between female and male HC (sGFAP: 67.8 vs. 67.5 pg/ml, *p* = 0.96; sNfL: 20.0 vs. 18.4 pg/ml, *p* = 0.93) and MOG-IgG^+^ patients (sGFAP: 92.2 vs. 64.1 pg/ml, *p* = 0.39; sNfL: 23.1 vs. 30.6 pg/ml, *p* = 0.66). sGFAP and sNfL were higher in male (sGFAP: 329.0 pg/ml; sNfL: 73.8 than female (sGFAP: 105.5 pg/ml, *p* = 0.17; sNfL: 21.1 pg/ml, *p* = 0.02) patients with AQP4-IgG^+^ NMOSD, but these evaluations were limited by the small number of male patients with AQP4-IgG^+^ NMOSD (*n* = 3).

### Baseline sGFAP and sNfL in patients with AQP4-IgG^+^ NMOSD, MOG-IgG^+^ patients, and healthy controls

**Fig. 2:**
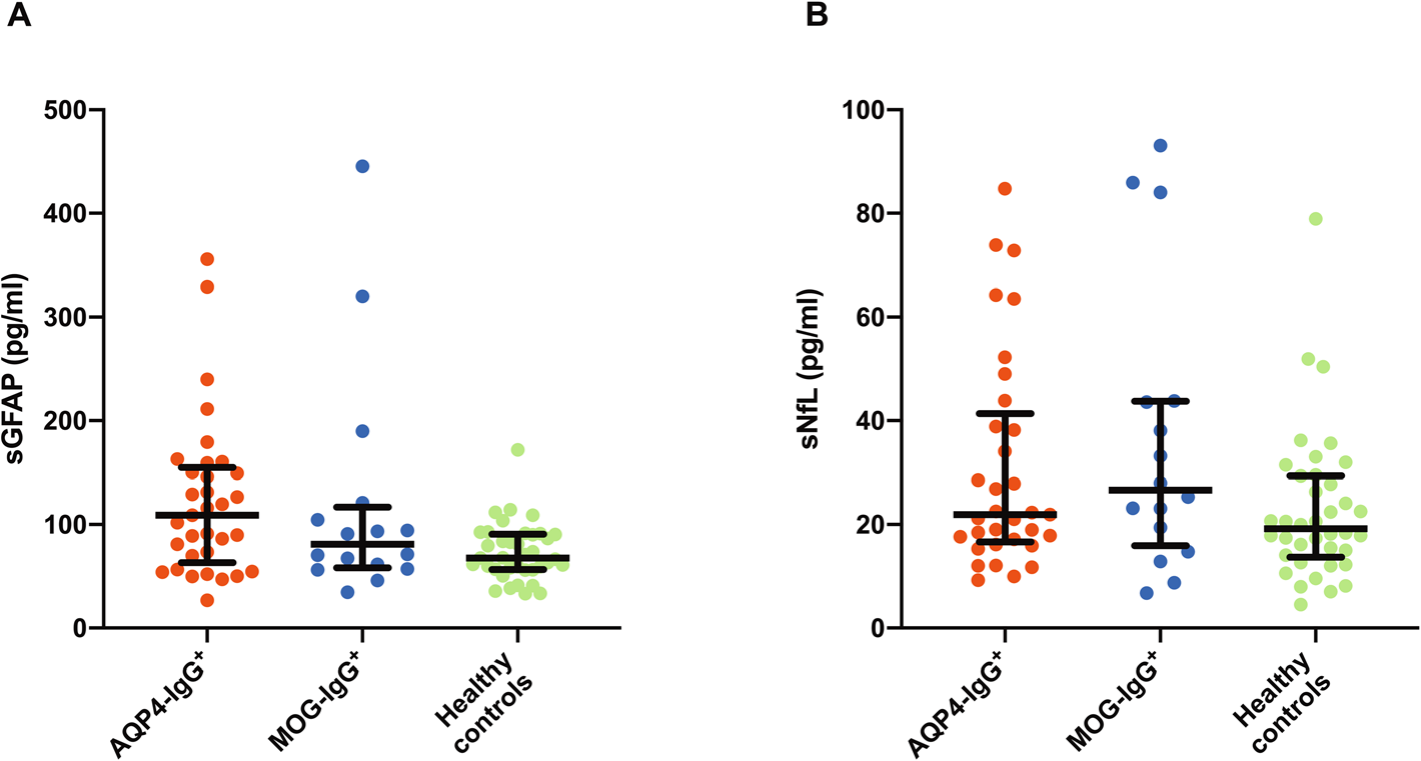
sGFAP and sNfL in patients with AQP4-IgG^+^ NMOSD, MOG-IgG^+^ patients, and healthy controls. Baseline sGFAP **a** and sNfL **b** of patients with AQP4-IgG^+^ NMOSD (*n* = 33), MOG-IgG^+^ patients (*n* = 16), and healthy controls (*n* = 38). Bars indicate median and interquartile range. *AQP4*-*IgG* aquaporin-4 immunoglobulin G, *MOG*-*IgG* myelin oligodendrocyte protein immunoglobulin G, *NMOSD* neuromyelitis optica spectrum disorder, *sGFAP* serum glial fibrillary acidic protein, *sNfL* serum neurofilament light chain protein

Median sGFAP levels were non-significantly higher in AQP4-IgG^+^ patients with NMOSD (109.2 pg/ml) than in MOG-IgG^+^ patients (81.1 pg/ml; *p* = 0.83) and HC (67.7 pg/ml; *p* = 0.07; Fig. 2a, Table 1). sNfL did not substantially differ between patients with AQP4-IgG^+^ NMOSD (21.9 pg/ml), MOG-IgG^+^ patients (26.6 pg/ml, *p* = 0.25), and HC (19.2 pg/ml, *p* = 0.69; Fig. 2b, Table 1).

### Association of sGFAP and sNfL with clinical disability scores at baseline

**Fig. 3:**
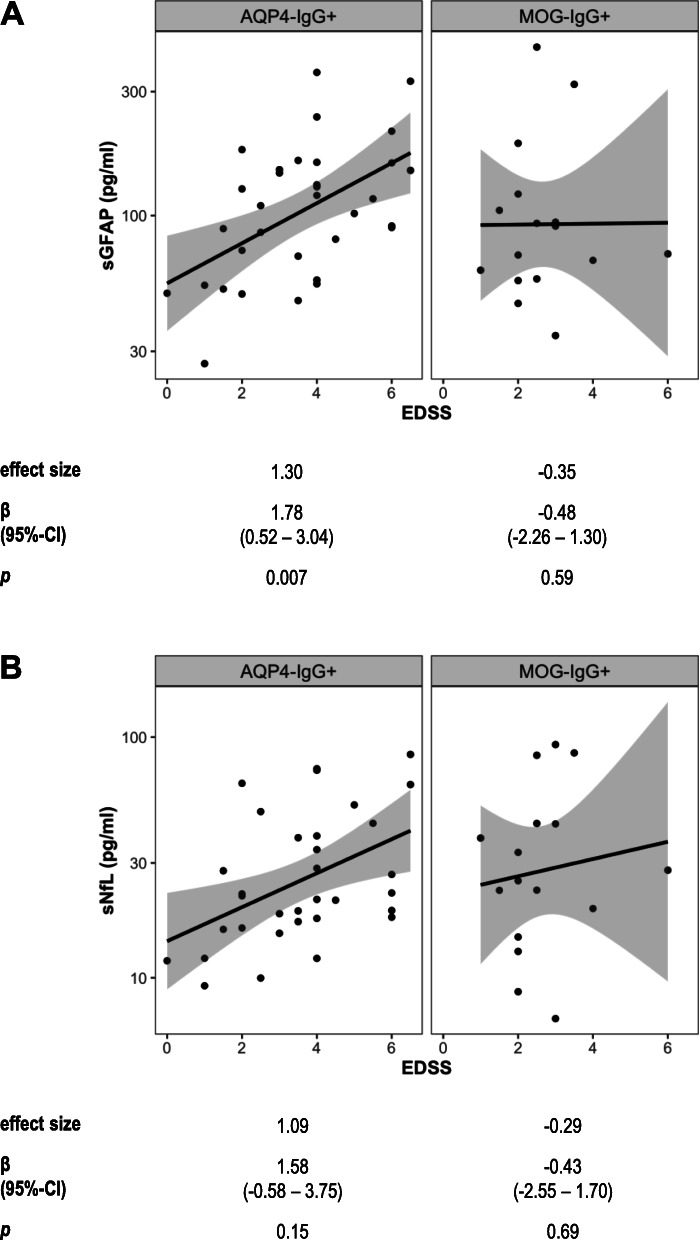
Association of sGFAP and sNfL with the EDSS. Association of sGFAP **a** and sNfL **b** with the Expanded Disability Status Scale (EDSS) score in AQP4-IgG^+^ (*n* = 33) and MOG-IgG^+^ (*n* = 16) patients. Results of linear models using log-transformed sGFAP and sNfL values adjusted for age and log-transformed time since last attack are shown below the plots. *AQP4*-*IgG* aquaporin-4 immunoglobulin G, *β* regression coefficient, *CI* confidence interval, *effect size* standardized effect size, *MOG*-*IgG* myelin oligodendrocyte protein immunoglobulin G, *NMOSD* neuromyelitis optica spectrum disorder, *sGFAP* serum glial fibrillary acidic protein, *sNfL* serum neurofilament light chain protein

We next evaluated whether sGFAP and sNFL are associated with widely used clinical disability scores, i.e., the EDSS and the MSFC, in AQP4-IgG^+^ as compared to MOG-IgG^+^ patients. Remarkably, higher sGFAP was strongly associated with a worse EDSS score in patients with AQP4-IgG^+^ NMOSD (SES = 1.30, *p* = 0.007), but not MOG-IgG^+^ patients (SES = − 0.35, *p* = 0.59; interaction effect of sGFAP by group η_p_^2^ = 0.10, *p* = 0.04; Fig. 3a, Table 2). There also was a non-significant association of higher sNfL with a worse EDSS score in AQP4-IgG^+^ (SES = 1.09, *p* = 0.15), but not MOG-IgG^+^ patients (SES = − 0.29, *p* = 0.69; interaction effect of sNfL by group η_p_^2^ = 0.06, *p* = 0.11; Fig. 3b, Table 2).

**Table 2:** Association of baseline sGFAP and sNfL with clinical disability parameters in AQP4-IgG^+^ and MOG-IgG^+^ patients

	Patient group (*n*)	sGFAP				sNfL			
		η_ρ_^2^ for interaction, *p*	Standardized effect size	β (95% CI)	*p*	η_ρ_^2^ for interaction, *p*	Standardized effect size	β (95% CI)	*p*
EDSS^a^	AQP4-IgG^+^ (33)	0.10, *p* = 0.04	1.30	1.78 (0.52–3.04)	0.007	0.06, *p* = 0.11	1.09	1.58 (− 0.58–3.75)	0.15
	MOG-IgG^+^ (16)		− 0.48	− 0.52 (− 2.26–1.30)	0.59		− 0.29	− 0.43 (− 2.55–1.70)	0.69
MSFC^a^	AQP4-IgG^+^ (25)	0.14, *p* = 0.03	− 1.28	− 0.73 (− 1.30 to − 0.16)	0.01	0.05, *p* = 0.20	− 1.75	− 1.05 (− 2.13−0.03)	0.06
	MOG-IgG^+^ (12)		0.76	0.43 (− 0.46–1.32)	0.33		− 0.37	− 0.22 (− 1.26–0.82)	0.67
9-HPT^a^	AQP4-IgG^+^ (32)	0.11, *p* = 0.04	− 1.03	− 0.007 (− 0.013 to − 0.001)	0.03	< 0.01, *p* = 0.70	− 0.82	− 0.006 (− 0.017–0.005)	0.28
	MOG-IgG^+^ (14)		0.65	0.004 (− 0.004–0.013)	0.32		− 0.47	− 0.003 (− 0.015–0.008)	0.56
PASAT^a^	AQP4-IgG^+^ (27)	0.05, *p* = 0.19	− 1.00	− 12.7 (− 25.1 to − 0.3)	0.045	0.13, *p* = 0.03	− 1.86	− 23.0 (− 43.7 to − 2.4)	0.03
	MOG-IgG^+^ (13)		0.21	2.7 (− 17.0–22.4)	0.78		0.44	5.5 (− 15.9–26.9)	0.61
T25-FW^b^	AQP4-IgG^+^ (30)	0.01, *p* = 0.61	0.19	0.027 (− 0.105–0.158)	0.69	0.01, *p* = 0.54	0.31	0.043 (− 0.179–0.265)	0.70
	MOG-IgG^+^ (14)		− 0.21	− 0.029 (− 0.212–0.154)	0.75		0.89	0.122 (− 0.099–0.343)	0.27

^a^Linear model using log-transformed sGFAP or sNfL values, including age as well as the log-transformed interval since the last attack as covariates. Furthermore, an interaction term of baseline sGFAP or sNFL (log-transformed) and group was included to assess the statistical significance of inter group differences

^b^Linear model using log-transformed sGFAP or sNfL and log-transformed T25-FW values, including age as well as the log-transformed interval since the last attack as covariates. Furthermore, an interaction term of baseline sGFAP or sNFL (log-transformed) and group was included to assess the statistical significance of inter group differences

Note that a higher EDSS score indicates a worse functional status, whereas a higher MSFC score indicates a better functional status. The EDSS [23] is the most common score to rate global neurological dysfunction secondary to MS and NMOSD. The MSFC [24] is a more complex, multidimensional scoring system for neurological impairment in MS and NMOSD, which consists of three components. These components, which may each be used individually as well, are the 9-HPT, PASAT, and T25-FW. The 9-HPT assesses upper extremity function and dexterity. PASAT, in rating the processing speed of auditory input and calculation ability, quantifies cognitive impairment. T25-FW addresses lower extremity function based on walking speed

*9*-*HPT* 9-hole peg test, *AQP4*-*IgG* aquaporin-4 immunoglobulin G, *β* regression coefficient, *CI* confidence interval, η_ρ_^2^ partial eta-squared, *EDSS* expanded disability status scale, *MOG*-*IgG* myelin oligodendrocyte protein immunoglobulin G, *MSFC* multiple sclerosis functional composite, *n* number, *NMOSD* neuromyelitis optica spectrum disorder, *PASAT* paced auditory serial addition test, *sGFAP* serum glial fibrillary acidic protein, *sNfL* serum neurofilament light chain protein, *T25*-*FW* timed 25-foot walk

Likewise, higher sGFAP was associated with a worse MSFC score in patients with AQP4-IgG^+^ NMOSD (SES = − 1.28, *p* = 0.01), but not MOG-IgG^+^ patients (SES = 0.76, *p* = 0.33; interaction effect of sGFAP by group η_p_^2^ = 0.14, *p* = 0.03; Table 2). There also was a non-significant association of higher sNfL with a worse MSFC score in AQP4-IgG^+^ (SES = − 1.75, *p* = 0.06), but not in MOG-IgG^+^ patients (SES = − 0.37, *p* = 0.67; interaction effect of sNfL by group η_p_^2^ = 0.05, *p* = 0.20; Table 2).

Analyses of the associations of sGFAP and sNfL with single components of the MSFC in AQP4-IgG^+^ and MOG-IgG^+^ patients showed that higher sGFAP was associated with worse performance in the 9-HPT in AQP4-IgG^+^ (SES = − 1.03, *p* = 0.03), but not MOG-IgG^+^ patients (SES = 0.65, *p* = 0.32; interaction effect of sGFAP by group η_p_^2^ = 0.11, *p* = 0.04). Higher sNfL was only non-significantly associated with worse 9-HPT in AQP4-IgG^+^ (SES = − 0.82, *p* = 0.28), but not MOG-IgG^+^ patients (SES = − 0.47, *p* = 0.56; interaction effect of sNfL by group η_p_^2^ < 0.01, *p* = 0.7; Table 2). Furthermore, higher sGFAP (SES = − 1.00, *p* = 0.045) and also sNfL (SES = − 1.86, *p* = 0.03) were associated with a worse PASAT score in AQP4-IgG^+^ patients with NMOSD. In contrast, neither sGFAP (SES = 0.21, *p* = 0.78) nor sNfL (SES = 0.44, *p* = 0.61) were associated with the PASAT score in MOG-IgG^+^ patients. Except for a non-significant positive association of sNfL with the T25-FW in MOG-IgG^+^ patients (SES = 0.89, *p* = 0.27), sGFAP or sNfL did not show any associations with the T25-FW in AQP4-IgG^+^ and MOG-IgG^+^ patients (Table 2).

When comparing AQP4-IgG^+^ patients with NMOSD treated (*n* = 29) or not (*n* = 4) with immunotherapy at baseline, median (IQR) sGFAP (101.8 [63.1–154.8] vs. 139.1 [67.5–157.5] pg/ml, *p* = 0.86) and sNfL levels (22.3 [16.0–41.4] vs. 20.1 [18.0–52.9] pg/ml, *p* = 0.48) did not significantly differ. In patients with AQP4-IgG^+^ NMOSD, neither sGFAP (SES = 0.35, *p =* 0.48) nor sNfL (SES = 0.14, *p* = 0.67) were associated with time on current immunotherapy. Because sGFAP and sNfL did not significantly differ in treated and untreated patients with AQP4-IgG^+^ NMOSD and given the low number of untreated AQP4-IgG^+^ patients (*n* = 4), we refrained from further comparative analyses of treated and untreated patients.

### sGFAP, sNfL, and clinical disability scores on follow-up

Clinical follow-up data as well as sGFAP and sNfL values were available from 24 patients with AQP4-IgG^+^ NMOSD at 1 year (IQR 12–14 months) after the baseline visit. In these patients, median (IQR) sGFAP and sNfL showed no substantial differences between baseline (median [IQR] sGFAP = 89.5 [54.1– 144.9] pg/ml; sNfL = 21.1 [16.0–42.4] pg/ml) and follow-up (sGFAP = 89.9 [57.8–148.3] pg/ml, *p* = 0.41; sNfL = 21.7 [14.7–41.5] pg/ml, *p* = 0.67). In addition, there were no significant differences in parameters of clinical disability (EDSS, MSFC, 9-HPT, PASAT, T25-FW) between baseline and 1-year follow-up (data not shown). Given the absence of substantial changes of sGFAP and sNfL values as well as of clinical disability parameters between baseline and 1-year follow-up, we refrained from analyses correlating baseline sGFAP and sNFL with clinical disability scores on follow-up and from analyses correlating changes of sGFAP and sNFL between baseline and follow-up with clinical disability scores. Furthermore, as only 3 of the 24 patients of whom baseline and follow-up sGFAP and sNfL values were available had one (2 patients) or two (1 patient) attacks between baseline and one year follow-up, we refrained from analyses correlating baseline and follow-up sGFAP and sNfL levels with intercurrent attacks.

### Association between sGFAP and sNfL at baseline and follow-up

**Fig. 4:**
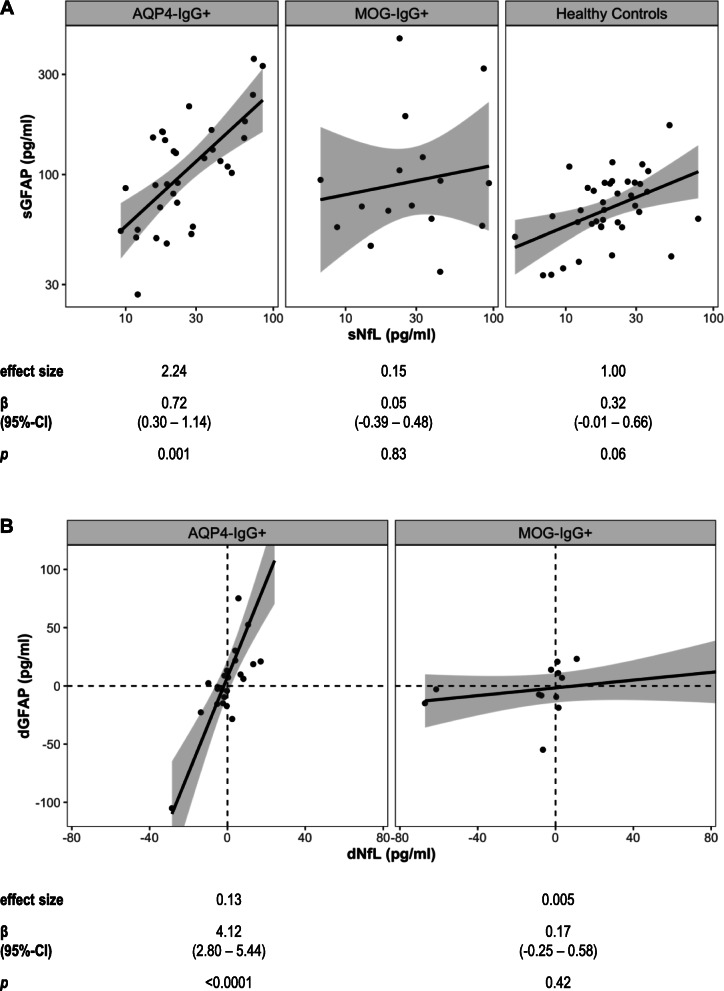
Associations between sGFAP and sNfL. **a** Association between log-transformed baseline sGFAP and sNfL in patients with AQP4-IgG^+^ NMOSD (*n* = 33), MOG-IgG^+^ patients (*n* = 16) and healthy controls (*n* = 38). **b** Association between changes in sGFAP (dGFAP) and sNfL (dNfL) concentrations, i.e., difference between one year and baseline values, in patients with AQP4-IgG^+^ NMOSD (*n* = 24) and MOG-IgG^+^ patients (*n* = 13). In Fig. 4b, one datapoint of an AQP4-IgG^+^ patient (dGFAP = 221.1 pg/ml, dNfL = 24.2 pg/ml) is not plotted for better visualization of datapoints with lower dGFAP, but included in the analyses. Results of linear models using log-transformed sGFAP and sNfL values adjusted for age and, where applicable (**b**), log-transformed time since last attack are shown below the plots. *AQP4*-*IgG* aquaporin-4 immunoglobulin G, *β* regression coefficient, *CI* confidence interval, *dGFAP* difference in sGFAP between one year and baseline, *dNfL* difference in sNfL between 1 year and baseline, *effect size* standardized effect size, *MOG*-*IgG* myelin oligodendrocyte protein immunoglobulin G, *NMOSD* neuromyelitis spectrum disorders, *sGFAP* serum glial fibrillary acidic protein, *sNfL* serum neurofilament light chain protein

Baseline sGFAP was clearly positively associated with baseline sNfL in patients with AQP4-IgG^+^ NMOSD (SES = 2.24, *p* = 0.001), but not in MOG-IgG^+^ patients (SES = 0.15, *p* = 0.83). In HC, baseline sGFAP and sNfL showed a non-significant positive association (SES = 1.00, *p* = 0.06; interaction effect of sNfL by group η_p_^2^ = 0.07, *p* = 0.049; Fig. 4). Changes in sGFAP and sNfL levels between baseline and one year (IQR 12–14 months) follow-up were positively associated in patients with AQP4-IgG^+^ NMOSD (SES = 0.13; *p* < 0.001), but not in MOG-IgG^+^ patients (SES = 0.005; *p* = 0.42; interaction effect of change in sNfL by group η_p_^2^ = 0.52, *p* < 0.0001; Fig. 4).

### Association of sGFAP and sNfL with disease duration and time since last attack

In patients with AQP4-IgG^+^ NMOSD, baseline sGFAP was neither associated with time since first manifestation of disease (SES = 0.18, *p* = 0.70) nor with time since last attack (SES = 0.41, *p* = 0.37; Table 3). In contrast, in patients with AQP4-IgG^+^ NMOSD, sNfL was non-significantly higher the shorter the time since first manifestation of the disease (SES = − 1.27, *p* = 0.08) and the shorter the time since last attack (SES = − 1.22, *p* = 0.10).

**Table 3:** Association of baseline sGFAP and sNfL with disease duration and time since last attack

sGFAP						sNfL			
Time between baseline visit and	Patient group (*n*)	η_ρ_^2^ for interaction, *p*	Std. effect size	β (95% CI)	*p*	η_ρ_^2^ for interaction, *p*	Std. effect size	β (95% CI)	*p*
First manifestation of disease^a^	AQP4-IgG^+^ (33)	0.05, *p* = 0.14	0.18	0.20 (− 0.84–1.24)	0.70	0.02, *p* = 0.40	− 1.27	− 1.37 (− 2.92–0.19)	0.08
	MOG-IgG^+^ (16)		− 0.96	− 1.10 (− 2.58–0.38)	0.14		− 1.99	− 2.14 (− 3.68 to − 0.60)	0.008
Last attack^a^	AQP4-IgG^+^ (33)	0.03, *p* = 0.25	0.41	0.46 (− 0.55–1.47)	0.37	< 0.01, *p* = 0.80	− 1.22	− 1.32 (− 2.88–0.25)	0.10
	MOG-IgG^+^ (16)		-0.47	− 0.53 (− 1.96–0.91)	0.46		− 0.99	− 1.08 (− 2.63–0.48)	0.17

^a^Linear models using log-transformed sGFAP and sNfL and time interval values, adjusted for age. Furthermore, an interaction term of baseline sGFAP or sNFL (log-transformed) and group was included to assess the statistical significance of inter group differences

*AQP4*-*IgG* aquaporin-4 immunoglobulin G, *β* regression coefficient, *CI* confidence interval, η_ρ_^2^ partial eta-squared, *MOG*-*IgG* myelin oligodendrocyte protein immunoglobulin G, *n* number, *NMOSD* neuromyelitis optica spectrum disorders, *sGFAP* serum glial fibrillary acidic protein, *sNfL* serum neurofilament light chain protein, *std*. *effect size* standardized effect size

In MOG-IgG^+^ patients, higher baseline sGFAP was non-significantly associated with shorter time since first manifestation of disease (SES = − 0.96, *p* = 0.14), but baseline sGFAP was not associated with time since last attack (SES = − 0.47, *p* = 0.46; Table 3). The non-significant negative association of sGFAP with time since first manifestation of disease in MOG-IgG^+^ patients should thus be interpreted with caution. However, in MOG-IgG^+^ patients, baseline sNfL was higher the shorter the time since first manifestation of disease (SES = − 1.99, *p* = 0.008) and there also was a non-significant association of higher baseline sNfL with shorter time since the last attack (SES = − 0.99, *p* = 0.17; Table 3).

### Association of baseline sGFAP and sNfL with future attacks in patients with AQP4-IgG^+^ NMOSD

**Fig. 5:**
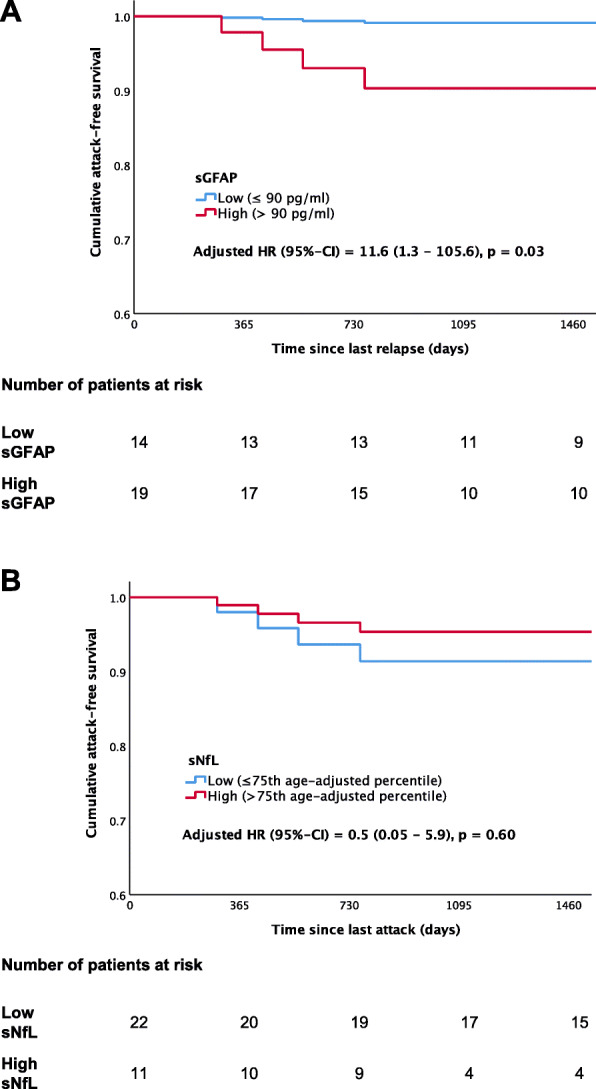
Association of baseline sGFAP and sNfL with time to a future attack in patients with AQP4-IgG^+^ NMOSD. **a** Patients with AQP4-IgG^+^ NMOSD (*n* = 33) were grouped into those with “high” and “low” baseline sGFAP values, using a sGFAP value of 90 pg/ml, derived from 75^th^ sGFAP percentile in healthy controls (90.7 pg/ml), as cut-off. The survival curves show the adjusted cumulative attack free survival probability in patients with baseline sGFAP ≤ or > 90 pg/ml. **b** Patients with AQP4-IgG^+^ NMOSD (*n* = 33) were grouped into those with “high” and “low” baseline sNfL values using the 75^th^ age-adjusted percentile as cut-off. The survival curves show the adjusted cumulative attack free survival probability in patients with baseline sNfL ≤ or > 75^th^ age-adjusted percentile. Hazard ratios (HR) with 95% confidence intervals (CI) calculated by Cox regression analyses adjusted for age and time since last attack prior to study inclusion and *p* values are indicated. The number of patients at risk for an attack in each group is indicated below the graphs. *CI* confidence interval, *NMOSD* neuromyelitis optica spectrum disorder, *HR* hazard ratio, *sGFAP* serum glial fibrillary acidic protein, *sNfL* serum neurofilament light chain protein

Data on attacks were available from all patients with AQP4-IgG^+^ NMOSD over a median (IQR) observation period of 51 (36–90) months. Among the 33 AQP4-IgG^+^ patients, 7 (21%) patients had at least one attack during the observation period. To evaluate an association of sGFAP with time to a future attack, patients were divided into “high” vs. ”low” sGFAP groups, using a baseline sGFAP level of 90 pg/ml, derived from the 75^th^ percentile of sGFAP in HC (90.7 pg/ml), as cut-off. In patients with AQP4-IgG^+^ NMOSD with baseline sGFAP > 90 pg/ml, the time to a first attack was shorter than in patients with baseline sGFAP ≤ 90 pg/ml (adjusted hazard ratio [HR] = 11.6, 95% CI = 1.3–105.6, *p* = 0.03; Fig. 5a). Of note, a similar percentage of patients was treated with immunotherapies in the “low” (93%) and “high” (84%, SMD = 0.27, *p* = 0.45) sGFAP groups. Similar results were obtained when using a baseline sGFAP value of 110 pg/ml, derived from the 90^th^ percentile of sGFAP in HC (109.4 pg/ml), as cut-off (> 110 pg/ml: *n* = 16, ≤ 110 pg/ml: *n* = 17, adjusted HR = 26.9, 95% CI = 2.0–360.8, *p* = 0.01). Due to the limited size of the MOG-IgG^+^ cohort, we refrained from analyses of associations of sGFAP and sNfL with future attacks in this group.

Based on data from a large HC cohort [30], we used the 75^th^ age adjusted sNfL percentile to dichotomize patients with AQP4-IgG^+^ NMOSD into “high” and ”low” baseline sNfL groups. The time to a new attack was similar in AQP4-IgG^+^ patients with “high” baseline sNfL compared to those with “low” baseline sNfL values (adjusted HR = 0.5, 95% CI = 0.05–5.9, *p* = 0.60; Fig. 5b). Again, a similar percentage of patients was treated with immunotherapies in the “low” (86%) and “high” (91%) sNfL groups (SMD = 0.14, *p* = 0.71). Similar results were obtained when using the 90^th^ age-adjusted sNfL percentile as cut-off (> 90^th^ age-adjusted percentile: *n* = 5, ≤ 90^th^ age-adjusted percentile: *n* = 28, adjusted HR = 9.0, 95% CI = 0.3–253.2, *p* = 0.20).

## Discussion

The key results of this detailed analysis of sGFAP and sNfL measured by Simoa in a well-characterized cohort of AQP4-IgG^+^ and MOG-IgG^+^ patients, most of which were in clinical remission at study baseline, as well as in HC are (1) sGFAP levels of AQP4-IgG^+^ patients were just mildly and not significantly higher than those of MOG-IgG^+^ patients and HC, and sNFL levels did not substantially differ between groups. (2) However, remarkably, sGFAP was still clearly associated with parameters of clinical disability (EDSS, MSFC) in patients with AQP4-IgG^+^ NMOSD. In contrast, no associations of sGFAP with parameters of clinical disability were observed in MOG-IgG^+^ patients. (3) While baseline sGFAP and sNfL were positively associated in patients with AQP4-IgG^+^ NMOSD, but not MOG-IgG^+^ patients, associations of sNFL with parameters of clinical disability in patients with AQP4-IgG^+^ NMOSD were weaker and less consistent than those of sGFAP. (4) In this prospective cohort with a median observation period of 4.25 years, higher baseline sGFAP, but not sNfL, was associated with a shorter time to a future attack in patients with AQP4-IgG^+^ NMOSD.

The median sGFAP levels of the 33 patients with AQP4-IgG^+^ NMOSD measured in this work (109.2 pg/ml) were lower than those reported by Watanabe et al. (207.7 pg/ml, *n* = 33 patients with NMOSD, 30 of which were AQP4-IgG^+^) and Kim et al. (123.1 pg/ml, *n* = 33 patients with AQP4-IgG^+^ NMOSD) [20, 21]. One may argue that this could be related to differences between Asian and Caucasian patients with AQP4-IgG^+^ NMOSD investigated in the previous studies and the present work. However, while serum samples were collected at a median of about 4 months after the last attack in both the studies of Watanabe et al. and Kim et al. [20, 21], the median interval between blood withdrawal and last attack in the AQP4-IgG^+^ patients studied in this work was 26 months. Both previous studies found higher sGFAP in patients with a recent attack than in patients in remission [20, 21]. The lower sGFAP levels seen in our work may thus very likely be explained by the longer interval between the last attack and serum withdrawal in the present study. Indeed, serial blood withdrawals in patients with mild to moderate traumatic brain injury showed that sGFAP peaks at 20 h after injury and thereafter declines over 72 h, indicating a relatively short half-life of sGFAP [31]. The lack of an association of sGFAP with time to last attack in patients with AQP4-IgG^+^ (Table 3) and the only mild elevation of sGFAP in patients with AQP4-IgG^+^ NMOSD as compared to MOG-IgG^+^ patients and HC (Fig. 2, Table 1) may therefore likewise be related to the long interval between the last attack and blood withdrawal. In contrast, longitudinal studies of patients with multiple sclerosis, traumatic brain injury, and stroke have shown that after increasing over days sNfL levels can remain elevated over months [32], which appears consistent with the associations of higher sNfL with shorter time to first manifestation of disease and shorter time to last attack in patients with AQP4-IgG^+^ NMOSD and MOG-IgG^+^ patients observed in the present work (Table 3). Altogether, our findings underscore a critical role of the timepoint of sGFAP determinations relative to a last attack when interpreting sGFAP values in patients with AQP4-IgG^+^ NMOSD.

Due to the low number of untreated AQP4-IgG^+^ patients with NMOSD included in our study, the absence of differences between sGFAP levels in treated and untreated patients with AQP4-IgG^+^ NMOSD should be regarded with caution. This study was not designed to detect influences of treatments on sGFAP levels and cannot exclude that differences in sGFAP may be detectable in larger studies specifically addressing the association of sGFAP with treatment status of patients with AQP4-IgG^+^ NMOSD.

The just mildly elevated sGFAP levels of patients with AQP4-IgG^+^ NMOSD in clinical remission do not suggest that sGFAP might be a useful diagnostic biomarker in this situation. However, our study confirms previously observed associations of GFAP in serum [20, 21] and CSF [13, 15] with the EDSS and additionally shows associations of sGFAP with the MSFC and some of its components (9-HPT, PASAT) in AQP4-IgG^+^ patients with NMOSD (Fig. 3, Table 2), suggesting a potential role for sGFAP as biomarker for disease severity in patients with AQP4-IgG^+^ NMOSD. Of note, the robust associations of higher sGFAP with worse clinical disability scores in the AQP4-IgG^+^ patients with NMOSD in clinical remission included in our study are rather remarkable, as they appear unlikely to be due to residual sGFAP elevations after a prior attack. While further research will be required to clarify the pathophysiological correlate of this finding, we propose two hypothetical explanations: First, sGFAP levels in AQP4-IgG^+^ patients with NMOSD in clinical remission could reflect ongoing subclinical astrocytic damage with consecutive GFAP release, possibly due to a persistent low level smoldering inflammation. Second, sGFAP levels in AQP4-IgG^+^ patients with NMOSD in clinical remission could reflect continuing astrocyte degeneration following an acute attack.

The association of higher sGFAP levels with a shorter time to a future attack in patients with AQP4-IgG^+^ NMOSD is an important finding of this study, suggesting that, if reproduced in independent prospective cohorts, sGFAP could be a biomarker for future disease activity in patients with AQP4-IgG^+^ NMOSD in clinical remission. However, while the sGFAP cut-off (90 pg/ml) established in the present work, which was based on the 75^th^ percentile of sGFAP in HC, may prove valid in patients with AQP4-IgG^+^ NMOSD with a long interval to the last attack, the generalizability of this cut-off needs to be further explored, and different cut-offs may apply to more active AQP4-IgG^+^ patient populations with a shorter interval to the last attack.

sNfL was not substantially increased in the investigated patient groups and, except for a negative association with the PASAT, only showed non-significant associations with disability markers in patients with AQP4-IgG^+^ NMOSD (Table 2). Furthermore, sNfL was not associated with future disease activity in AQP4-IgG^+^ NMOSD (Fig. 5). These findings suggest that sGFAP is more specifically and more strongly associated with the disease process of AQP4-IgG^+^ NMOSD than sNfL, which complies well with the pathophysiological concept of AQP4-IgG^+^ NMOSD being an antibody-mediated astrocytopathy [5, 6]. The positive associations of sGFAP and sNfL in AQP4-IgG^+^ NMOSD, but not MOG-IgG^+^ patients (Fig. 4), therefore seem compatible with a scenario in which a primary antibody-mediated astrocytopathy results in secondary neuroaxonal damage. In this scenario, sNfL would only be indirectly linked to the disease process of AQP4-IgG^+^ NMOSD, which might explain the weaker or absent associations of sNfL with clinical disability parameters and future disease activity in AQP4-IgG^+^ patients. Altogether, our present findings rather argue against a role of sNfL as disease severity or prognostic biomarker in AQP4-IgG^+^ NMOSD in phases of clinical remission.

From a clinical practice point of view, blood-based biomarkers would be particularly useful in patients with AQP4-IgG^+^ NMOSD in clinical remission, where decisions on continuation, escalation or de-escalation of relapse preventing therapies have to be made, which sometimes can be clinically challenging. The associations of sGFAP with clinical disability and the potential prognostic value of sGFAP in patients with AQP4-IgG^+^ NMOSD in phases of clinical remission seen in the present study overall suggest that further investigations on the value of sGFAP measurements in independent cohorts of patients with AQP4-IgG^+^ NMOSD in phases of clinical remission are warranted. Such studies may also evaluate the potential role of sGFAP to guide treatment decisions in patients with AQP4-IgG^+^ NMOSD.

Advantages of this study are its prospective design with a long observation period and the highly standardized acquisition of serum samples and comprehensive clinical data. Nevertheless, although the number of patients with AQP4-IgG^+^ NMOSD analyzed in this work was similar to that of previous studies [20, 21], one limitation of this monocentric work is the number of AQP4-IgG^+^ and MOG-IgG^+^ patients, which both are rare disease entities, available for analysis.

## Conclusions

This study suggests a potential role for sGFAP as biomarker for disease severity and future disease activity in patients with AQP4-IgG^+^ NMOSD in phases of clinical remission. This is consistent with the pathophysiological concept of AQP4-IgG^+^ NMOSD being an immune-mediated astrocytopathy. The potential relevance of sGFAP as disease severity and prognostic biomarker in AQP4-IgG^+^ NMOSD thus warrants to be further explored in independent cohorts of AQP4-IgG^+^ patients with NMOSD.

## Data Availability

The datasets generated and/or analyzed during the current study are not publicly available due to local regulations concerning protection of patient data, but on reasonable requests, approval for distribution of data will be obtained by the institutional review board of Charité—Universitätsmedizin Berlin and anonymized data will be made available by the corresponding author.
